# Screening for Forensically Relevant Drugs Using Data-Independent High-Resolution Mass Spectrometry

**DOI:** 10.1021/acs.chemrestox.3c00379

**Published:** 2024-04-04

**Authors:** Maia N. Bates, Abby E. Helm, Heather M. Barkholtz

**Affiliations:** † Department of Chemistry, College of Letters and Science, University of Wisconsin-Madison, 1101 University Avenue, Madison, Wisconsin 53706, United States; ‡ Forensic Toxicology Section, Environmental Health Division, Wisconsin State Laboratory of Hygiene, 2601 Agriculture Drive, Madison, Wisconsin 53718, United States; § Pharmaceutical Sciences Division, School of Pharmacy, University of Wisconsin-Madison, 777 Highland Avenue, Madison, Wisconsin 53705, United States

## Abstract

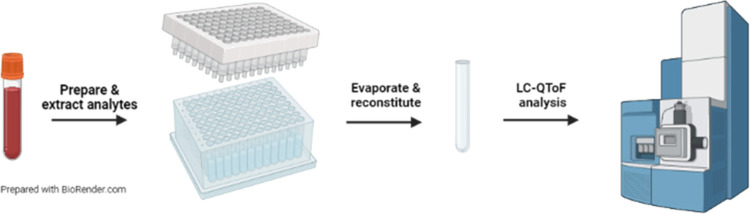

Forensic and clinical laboratories are expected to provide a rapid screening of samples for a wide range of analytes; however, the ever-changing landscape of illicit substances makes analysis complicated. There is a great need for untargeted methods that can aid these laboratories in broad-scope drug screening. Liquid chromatography hyphenated with high-resolution mass spectrometry (LC-HRMS) has become a popular technique for untargeted screening and presumptive identification of drugs of abuse due to its superior sensitivity and detection capabilities in complex matrices. An untargeted extraction and data acquisition method was evaluated for the broad screening of high-priority drugs of abuse in whole blood. A total of 35 forensically relevant target analytes were identified and extracted at biologically relevant low and high (10× low) concentrations from whole blood using supported liquid extraction. Data-independent acquisition was accomplished using ultraperformance liquid chromatography and a quadrupole time-of-flight mass spectrometry. Results were acceptable for screening assays, with limits of detection at or below the recommended low-concentration cutoffs for most analytes. Analyte ionization varied from 30.1 to 267.6% (average: 110.5%) at low concentrations and from 8.6 to 383.5% (average: 93.6%) at high concentrations. Extraction recovery ranged from 8.5 to 330.5% (average: 105.3%) at low concentrations and from 9.4 to 127.5% (average: 82.7%) at high concentrations. This variability was also captured as precision, ranging from 4.7 to 135.2% (average: 36.5%) at low concentrations and from 0.9 to 59.0% (average: 21.7%) at high concentrations. The method described in this work is efficient and effective for qualitative forensic toxicology screening, as demonstrated by analysis of 166 authentic suspected impaired driver and postmortem specimens. That said, it is critical that laboratories establishing untargeted LC-HRMS screening assays be aware of the strengths and limitations across diverse drug categories and chemical structures.

## 1 Introduction

From 2018 to 2022, 218 novel psychoactive substances were identified by the Center of Forensic Science Research and Education’s novel psychoactive substance (NPS) Discovery team in the United States (US).^1^ Of these new substances, opioids, stimulants, and cannabinoids represent the largest subclasses. Similarly, the US Centers for Disease Control and Prevention (CDC) found that over 80% of overdose deaths in 2019 involved methamphetamine, cocaine, and/or opioids.^2^ As the number of drugs and metabolites that may be present in a sample increases, forensic and clinical laboratories must employ broad screening methods to detect a wide range of analytes in one assay.^3^ The landscape of drugs of abuse and toxins includes diverse drug classes and chemical properties, which has posed the greatest challenge for broad screening in routine toxicological analysis.

With long lists of drugs to screen for, identifying highest-priority analytes and standardization across laboratories is difficult. Recent surveys of drug testing practices in driving under the influence of drug (DUID) investigations from laboratories across the US and Canada were reviewed by the National Safety Council’s (NSC’s) Alcohol, Drugs, and Impairment Division to provide recommendations for the scope and sensitivity of drug testing. These recommendations are regularly reevaluated and updated to reflect the changes in the drug landscape and have been a great resource for toxicological laboratories after the first document was published in 2007.^4^ This process of surveying laboratories and producing scope of testing and sensitivity recommendations was updated in 2017^5^ and again in 2021.^6^ To further promote standardization across laboratories, the American Academy of Forensic Sciences Standards Board (ASB), an accredited standards development organization, adopted ASB Standard 120 “Standard for the Analytical Scope and Sensitivity of Forensic Toxicology Blood Testing in Impaired Driving Investigations”.^7^ This standard was derived from the NSC’s 2017 recommendations.^5^

Current methods for screening a range of analytes typically rely on a targeted multianalyte approach.^3,8^ Immunoassays are commonly used in clinical and forensic laboratories for drug screening in urine due to their specificity, ease of use, and rapid results.^9^ However, immunoassays often miss new or less common substances. There is also the need for multiple immunoassays for the different drug classes, requiring several tests. Gas chromatography–mass spectrometry (GC-MS) is another common screening method employed in urine toxicology. GC-MS offers the advantage of untargeted analysis but has inherent disadvantages of limited sensitivity, prolonged run times, and often requiring derivatization steps which can be costly and time-consuming.^10,11^ Alternatively, liquid chromatography–tandem mass spectrometry (LC-MS/MS) techniques have emerged as the preferred toxicological screening method for urine and other biological matrices, such as blood. Using LC in place of GC conserves the polarity, thermally labile, etc. compounds in the mobile phase.^12^ Separation is achieved through the exploitation of differences in analyte chemical properties and solubility by adjusting the mobile phases over the course of separation. Furthermore, LC-MS/MS instrument technology improvements have allowed for the faster, more efficient separation of more analytes. Particularly ultrahigh-performance liquid chromatography (UHPLC) demonstrates higher pressures and smaller particle sizes to achieve higher resolution, faster run times, and less solvent usage than the standard high-performance liquid chromatography (HPLC) techniques many laboratories are familiar with.^12^

Another consideration that toxicological laboratories must make for comprehensive screening is the method of data acquisition. High-resolution mass spectrometry (HRMS) offers advanced means of identification through increased sensitivity, exact monoisotopic mass, and distinctive fragmentation.^8^ The use of HRMS for toxicological screening is not new. Typically, a targeted approach is taken using data-dependent acquisition (DDA) techniques, such as multiple reaction monitoring (MRM). This works well when looking for and quantifying common substances, but this approach falls short for analytes that are not preselected for fragmentation. Any information on unknown substances will be missed.^8^

In response, there have also been efforts to explore other means of acquiring and processing the HRMS data accumulated during screening. An alternate approach includes data-independent acquisition (DIA) where a nontargeted screen of precursor ion mass is performed, and the most abundant ions are selected for further fragmentation and identification from a spectral library.^13^ This acquisition method is more ideal for screening than DDA as data collection is analyte agnostic, but this process is still limited concerning low abundance ions such as high potency substances (e.g., fentanyl and its analogues).^14^ Sequential window acquisition of all theoretical fragment ion spectra (SWATH) was developed as a potential solution to the limitations of DDA and DIA. SWATH operates by analyzing “mass windows” where precursor ions within a small mass range undergo fragmentation and detection before moving on to the next mass window.^15^ This acquisition is much closer to an untargeted acquisition approach; however, the limitations lie in shared product ions of drugs in similar classes and complexity of the data generated as well as limited instruments with the capability to perform SWATH data acquisition.^16,17^

The most promising approach to wide-scope analysis in recent years has been UHPLC-HRMS with untargeted data acquisition, such as DIA. HRMS on an instrument such as a quadrupole time-of-flight (QToF) mass spectrometer offers high sensitivity for low concentrations of analytes in complex mixtures and DIA which is well suited for flexible screening and adjustments to the spectral library used for matching as all parent ions are selected for fragmentation and maximum information about a sample is gained.^18,19^ Success using this technique for drug abuse screening has been well documented with different approaches, such as extraction techniques. A study from 2012 by Birkler et al. utilized solid phase extraction (SPE) cartridges for extracting 46 common drugs of abuse from whole blood.^18^ Around the same time, Guale et al. developed an automated SPE approach to identify several forensically relevant drugs and NPS.^19^ In a similar study by Partridge et al. in 2018, a liquid–liquid extraction (LLE) method was used to extract analytes from whole blood followed by HRMS for forensic screening.^20^ The work done in these studies further highlights the multitude of approaches one can take to broad toxicological screening. More recently Ayala et al. sought to develop a method for screening of common drugs of abuse, NPS, and cannabinoids from whole blood using a supported liquid extraction technique followed by LC-QToF-MS analysis.^21^ There was some success with this extraction technique, particularly with cannabinoids, which are often left out of broad screening target lists. Similarly, Goh et al. developed an LC-QToF-based qualitative assay specifically targeting synthetic cannabinoids, cathonines, and their metabolites in urine.^22^

While there have been many efforts in the toxicology field to address these shortcomings, there is still a lack of one extraction and data acquisition method that works well for all analytes and can meet the high-throughput demands of a toxicology laboratory.^23−26^ The aim of this work was to evaluate an untargeted workflow for broad toxicological screening of the most recent Tier I drugs of abuse and metabolites.^6^ The proposed method includes the untargeted extraction of analytes at biologically relevant concentrations from whole blood. Method performance measures included those required by ABS Standard 036 “Standard Practices for Method Validation in Forensic Toxicology”^27^ for qualitative methods (i.e., interference, carryover, and limit of detection). To probe deeper into the strengths and weaknesses of this method, the extraction recovery, ion suppression or enhancement, and precision were also assessed. To do this, we utilized supported liquid extraction (SLE) and UPHLC-HRMS with DIA to provide clinical and forensic laboratories a quick, adaptable, and high-throughput screening procedure.

## 2 Materials and Methods

### 2.1 Chemicals and Reagents

Whole blood used in this work was pooled from multiple donors, sourced from the American Red Cross. The pooled blood is filtered, preserved with sodium fluoride at a concentration of approximately 1 g/100 mL (1% w/v), and stored at 2–8 °C. The preserved blood was screened for the presence of potentially interfering pharmaceuticals, drugs, and metabolites via LC-QToF-MS analysis. Results were compared to an in-house spectral library containing over 800 drugs of abuse, pharmaceuticals, and their metabolites.

All standards were purchased from Cayman (Ann Arbor, MI). Stock solutions were prepared in HPLC-grade methanol (MeOH). HPLC-grade acetonitrile (ACN), HPLC-grade MeOH, and ammonium formate were acquired from Thermo Fisher Scientific (Bridgewater, NJ). Ammonium formate and formic acid (98% w/w) were acquired from Sigma-Aldrich (Burlington, MA). Working standard solutions were prepared by diluting stock standards in 1:1 (by volume, or v/v) water:methanol at various concentrations and stored at −20 °C until use. All analytes and concentrations considered here are listed in Table 1. A deuterated internal standard (ISTD) solution was prepared using (±)-warfarin-d5 diluted in methanol to a concentration of 1 μg/mL and stored at −20 °C. For analysis, the ISTD was diluted to a final concentration of 1 ng/mL in HPLC-grade methanol. Purified water was prepared in-house by using an Elga PURELAB Ultra water purification system.

**Table 1:** Method Performance Parameters for the 35 NSC Tier I Drugs and Metabolites Considered in This Work^a^

analyte	concentration (ng/mL)	ionization, % (SD, %)	recovery, % (SD, %)	precision % (SD, %)
stimulants				
methamphetamine	10	94.3 (30.5)	70.6 (8.4)	29.8
	100	66.8 (15.4)	64.9 (1.0)	24.1
amphetamine	10	-	-	-
	100	67.3 (9.7)	74.6 (3.9)	16.5
cocaine	20	255.7 (148.0)	110.4 (2.4)	57.5
	200	139.9 (39.8)	92.2 (1.0)	28.8
cocaethylene	10	107.4 (28.6)	102.1 (2.5)	25.2
	100	89.3 (23.5)	85.7 (0.8)	26.5
benzoylecgonine	50	173.2 (31.6)	99.7 (2.8)	17.8
	500	113.7 (21.6)	91.2 (1.4)	18.9
3,4-methylenedioxy	10	30.1 (16.9)	82.6%(15.7)	54.4
methamphetamine (MDMA)	100	86.9 (12.3)	84.8 (4.6)	15.3
3,4-methylenedioxy	10	122.8 (17.5)	155.3 (16.1)	14.8
amphetamine (MDA)	100	99.9 (25.6)	115.5 (5.6)	27.3
depressants				
carisoprodol	1000	47.4 (14.9)	128.7 (5.9)	30.1
	10000	50.3 (20.1)	12.4 (0.7)	41.5
meprobamate	2000	267.6 (163.6)	60.9 (121.7)	59.9
	20000	310.2 (182.1)	13.8 (0.6)	59.0
zolpidem	10	37.3 (20.5)	60.6 (121.7)	54.50
	100	54.3 (15.1)	57.5 (0.7)	25.8
benzodiazepines				
7-aminoclonazepam	10	70.3 (3.7)	94.9 (3.0)	4.7
	100	73.1 (3.2)	101.5 (3.3)	2.2
α-hydroxyalprazolam	10	130.8 (11.9)	115.7 (5.2)	7.5
	100	104.2 (5.0)	126.7 (3.3)	3.7
alprazolam	10	89.9 (17.7)	143.4 (5.7)	21.7
	100	93.6 (16.2)	127.5 (2.5)	15.3
clonazepam	10	211.4 (288.2)	107.0 (4.6)	135.2
	100	71.2 (10.4)	107.7 (2.7)	15.6
lorazepam	10	-	-	-
	100	74.2 (10.0)	92.8 (2.4)	14.5
diazepam	10	54.1 (7.1)	68.7 (0.5)	13.8
	100	65.6 (13.1)	79.6 (3.0)	17.8
nordiazepam	10	111.1 (10.4)	93.3 (2.1)	9.5
	100	92.8 (1.9)	95.4 (2.9)	2.5
oxazepam	10	82.6 (33.6)	79.7 (7.5)	36.2
	100	81.9 (28.9)	101.0 (4.3)	32.2
temazepam	10	108.9 (16.2)	111.3 (5.4)	18.1
	100	83.7 (9.2)	109.1 (3.1)	10.7
narcotic analgesics				
codeine	10	114.9 (96.9)	170.3 (214.0)	93.4
	100	50.6 (8.9)	60.8 (3.2)	18.6
6-acetylmorphine (6-AM)	4	41.1 (9.9)	8.5 (2.1)	26.1
	40	383.5 (53.8)	103.3 (9.7)	17.40
hydrocodone	10	108.9 (96.4)	192.3 (188.6)	29.7
	100	51.2 (7.5)	88.2 (6.3)	16.9
hydromorphone	10	71.1 (51.7)	54.1 (4.9)	84.7
	100	34.2 (9.8)	39.4 (10.8)	30.0
morphine	10	33.0 (34.3)	86.4 (140.9)	41.9
	100	8.6 (4.1)	9.4 (0.9)	42.9
oxycodone	10	258.4 (210.1)	330.5 (448.0)	23.7
	100	133.1 (32.4)	105.6 (9.8)	24.7
oxymorphone	10	-	-	-
	100	-	-	-
fentanyl	1	84.9 (26.9)	113.0 (5.8)	30.6
	10	66.7 (16.4)	88.3 (0.8)	25.1
buprenorphine	1	50.4 (21.2)	40.7 (12.9)	19.3
	10	60.6 (41.7)	86.6 (3.9)	15.8
norbuprenorphine	1	213.6 (195.3)	99.2 (29.9)	79.9
	10	90.0 (24.1)	102.6 (4.1)	26.3
methadone	20	78.7 (20.9)	102.5 (5.2)	29.8
	200	65.8 (20.5)	85.6 (4.8)	31.6
tramadol	50	74.1 (3.4)	83.4 (2.4)	4.8
	500	65.8 (1.1)	77.5 (1.5)	0.9
o-desmethyltramadol	50	79.7 (4.9)	87.4 (3.0)	6.3
	500	71.0 (0.5)	83.6 (0.8)	0.9
cannabinoids				
Δ^9^-tetrahydrocannabinol (Δ^9^-THC)	5	-	-	-
	50	-	-	-
11-hydroxy-Δ^9^-tetrahydrocannabinol (OH-Δ^9^-THC)	5	-	-	-
	50	-	-	-
11-nor-9-carboxy-Δ^9^-tetrahydrocannabinol (COOH-Δ^9^-THC)	5	119.3 (46.4)	289.8 (130.9)	22.8
	50	109.5 (26.3)	178.5 (26.7)	14.4

a Results include the low and high concentrations considered here as well as results for ion suppression or enhancement, recovery, and precision. Standard deviation (SD) is reported for ionization and recovery results, displayed as gray font in parentheses behind average values. Dashes indicate that the analyte was not reliably detected at that concentration.

### 2.2 Sample Extraction

Whole blood samples were fortified with NSC’s Tier 1 drugs of abuse at the concentrations at or below those recommended as screening low concentration cutoffs (herein called “low”) as well as 10× that (herein called “high”) and analyzed.^6^ Most recent NSC Tier I drugs and metabolites screening recommendations include suggested low-concentration cutoffs for screening and confirmation testing in various biological fluids.^6^ Screening low-concentration cutoffs are given for 12 of the 35 analytes (methamphetamine, amphetamine, benzoylecgonine, carisoprodol, zolpidem, burprenorphine, fentanyl, methadone, morphine, oxycodone, tramadol, and 11-nor-9-carboxy-Δ^9^-tetrahydrocannabinol). Confirmation of low-concentration cutoffs is given for all analytes except for α-hydroxyalprazolam. A summary of the recommended screening low-concentration cutoffs for blood specimens is included in Supporting Table S1, as well as the concentrations considered in this work. These screening cutoffs are chosen by the NSC to accommodate laboratories that rely solely on immunoassays for presumptive screening as immunoassays do not have the same level of sensitivity as LC-MS/MS or LC-HRMS technology.^6^

Aliquots of the fortified blood or authentic specimens (100 μL) were pipetted into a 96-well Agilent Captiva Enhanced Matrix Removal-lipid cleanup plate fitted over a 96-well collection plate filled with glass inserts, followed by 10 μL of internal standard and allowed to equilibrate for 5 min. A crashing solvent of ice-cold 15:85 (v/v) MeOH:ACN (400 μL) was then added, and the whole plate was vortexed. A Waters 96-well positive-pressure manifold was used for elution by starting with low pressure, 0.5–1 psi, and increasing slowly up to 15 psi. Next, 1:4 (v/v) MeOH:H_2_O (200 μL) was added, and the samples were vortexed and eluted again following the procedure described above until the extraction plate was dry. The eluted samples in the glass inserts were dried using an Organomation microplate evaporator (N_2_, 30 °C) and reconstituted with a 1:1 (v/v) MeOH:H_2_O solution to achieve a final volume of 150 μL. Finally, the samples were vortexed, centrifuged for 5 min at 3000 rpm, and placed in the instrument’s sample manager for analysis.

### 2.3 Instrument Parameters

Ultrahigh-performance chromatographic separation was performed on a Waters Acquity HSS C_18_ column (2.1 mm × 150 mm, 1.8 μm particles) maintained at 50 °C. All gases were set up according to the manufacturer’s specifications. Samples were maintained at 15 °C. The injection volume was 5 μL, and the liquid flow rate was set to 0.400 mL/min. Mobile phases consisted of *A*_1_ = 5 mM ammonium formate at pH 3.0, *B*_1_ = acetonitrile and 0.1% formic acid, *A*_2_ = water and 0.001% formic acid, and *B*_2_ = acetonitrile and 0.001% formic acid. Chromatographs were collected over a 15 min period in positive acquisition mode, and the mobile phases consisted of a gradient *A*_1_ and *B*_1_. To start, 13.0% *B*_1_ for 10 min, followed by 50% of each mobile phase for 0.75 min, then 95% *B*_1_ for 1.5 min, and back to original conditions for the rest of the 15 min period. Data was collected over a 7.5 min period in negative-ion acquisition mode, and the mobile phases consisted of a gradient of *A*_2_ and *B*_2_. To start, 13% *B*_2_ for 4.5 min, then 95% *B*_2_ for 1 min, and back to original conditions for the remaining time.

A Waters Xevo G2-XS QToF mass spectrometer with an electrospray ionization (ESI) source was used for detection in positive and negative ionization modes. Supporting Table S2 contains the ESI mode, expected retention times, neutral mass, exact mass, and fragments for each analyte. Positive ion mode conditions were set as follows: capillary voltage of 0.80 kV, sample cone at 25 V, and cone gas flow set to 20 L/h. Negative-ion mode conditions were as follows: capillary voltage of 1.50 kV, sample cone at 40 V, and cone gas flow set to 50 L/h. Source temperature and desolvation temperature were consistent for both modes at 150 and 400 °C, respectively, and desolvation gas flow was set to 800 L/h.

Data-independent acquisition mode was used with three MS functions (MS^E^).^28^ A low collision energy of 6 eV was applied to limit fragmentation. This was followed by a ramped high collision energy of 10–40 eV to generate the maximum information from fragment ions. Precursor and fragment ion data was collected from 40 to 1000 *m/*z. Finally, lock mass data was acquired for online mass calibration. The lock mass is a known reference mass that is periodically infused during analysis to provide an exact mass calibration. For this experiment, the reference solution was leucine enkaphalin (commonly known as LeuEnk).^29^ Peak detection was performed using the Waters UNIFI three-dimensional (3D) peak algorithm, and *m/z-*retention time pairs were matched against an in-house spectral library.

### 2.4 Authentic Samples

Authentic suspected impaired driver and postmortem whole blood samples (*N* = 166) submitted to the Wisconsin State Laboratory of Hygiene (WSLH) were included in this work. Specimens were received by the WSLH Forensic Toxicology Section as part of normal business. Once specimens had exceeded their record retention windows and were ready to be discarded, residual volume was aliquoted into new containers and deidentified. Use of deidentified residual human specimens in this project was approved by the University of Wisconsin-Madison’s IRB under Protocol ID number 2023–0608-CP002. Due to the deidentification process, no demographic details were retained nor any information on the submitting agency (e.g., suspected impaired driver versus post-mortem). Authentic samples then underwent the sample extraction and LC-QToF-MS data collection methods described above. Extracts were analyzed in both positive- and negative-ion modes. Data analysis was limited to analytes included in this work (i.e., NCS’s Tier I drugs and metabolites).

### 2.5 Data Analysis

An in-house library composed of over 800 compounds containing mainly drugs of abuse, pharmaceuticals, and their metabolites was used for in-house spectral matching through the UNIFI 3D peak detection software. Our in-house library includes the Commercial Waters Toxicology Library version 1.9 and additional drugs of abuse and metabolites from reference materials or monograph information. Waters UNIFI Scientific Information System (version 9) software was used to acquire, process, and visualize data. Spectral matches were accepted using the following criteria: exact mass match within ±5 ppm of expected, proper retention time based on method specifications (in this case, ± 0.1 min of expected RT), and all expected fragment ions (see Supporting Table S2) are present when compared to reference spectra. Microsoft Excel Professional Plus 2016 was used to coalesce and process the method performance measures, as described below.

### 2.6 Method Performance Measures

Performance of the outlined method was measured using these criteria: limits of detection, both endogenous and exogenous interferents, carryover, recovery, ion suppression or enhancement, and precision. ABS Standard 036 requires that validation of qualitative methods include, at minimum, interferent, carryover, and limit of detection studies.^27^ To gain a deeper understanding of the strengths and weaknesses of this wide-scope, data-independent acquisition approach, measures of extraction recovery, ion suppression or enhancement, and precision were also considered. Processed sample stability and carryover from concentrations higher than what was included here are outside the scope of this work. Each analyte was considered at a low and high (10× low) concentration, as detailed in Supporting Table S1. Analyte recovery, ion suppression or enhancement, and precision were found at both low and high concentrations. To correct for variations in the sample preparation and data acquisition steps, all analyte data was normalized to an internal standard (ISTD), (±)-warfarin-d5. Detector counts, or “peak area” of each analyte and the ISTD were used to normalize data and calculate all performance measures described below.

#### 2.6.1 Limit of Detection (LOD)

One of the advantages of using high-resolution mass spectrometry for analysis is the high sensitivity for the detection of low concentrations of analytes.^30^ The LODs within this experimental setup were determined by using scalar dilutions of the target analytes fortified in whole blood and extracted. As described by ASB Standard 036 for Using Decision Point Concentrations as the LOD,^6^ at least three blank blood lots were fortified with the analyte at the low concentrations considered here and assessed over at least three runs. That is, dilutions continued until the low concentration considered in this work (listed in Table 1) were reached, and all samples were analyzed in triplicate. Results are reported for which analyte LODs were above the low concentrations considered in this work.

#### 2.6.2 Carryover

Carryover of analytes from sample to sample was evaluated by injecting blanks containing 1:1 (v/v) MeOH:H_2_O after the high concentration considered in this work for each analyte. Blanks were assessed for the presence of target analytes.^31^

#### 2.6.3 Interferents

Endogenous interferents were assessed via fortifying several lots of pooled whole blood with target analytes. Exogenous interferents were considered as well by fortifying whole blood with mixtures of analytes with similar structures and/or properties,^32^ see Supporting Table S3. Special emphasis was placed on analytes frequently observed in routine casework (e.g., Δ^9^-tetrahydrocannabinol, methamphetamine, amphetamine, fentanyl, etc.) and closely eluting isomers (hydromorphone and morphine).

#### 2.6.4 Ion Suppression or Enhancement

Ion suppression or enhancement is particularly important when evaluating multiple analytes at low concentrations in complex matrices,^33,34^ such as this work. Ion suppression or enhancement experiments were performed in two steps. First, six replicates of blank blood samples fortified with analytes at their low and high concentrations were extracted as described in Section 2.2. Second, six replicates of blank blood samples were extracted as described in Section 2.2 followed by fortification of the extract with analytes at their low and high concentrations. The peak area detector counts of an extracted sample (“peak area extracted analyte”) were compared to the peak area of an analyte fortified after extraction (“peak area fortified extract”) using eq 1. A value below 100% indicated ion suppression, and a value greater than 100% indicated ion enhancement.
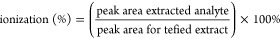
1

#### 2.6.5 Analyte Recovery

Analyte recovery (extraction efficiency) was evaluated in quadruplicate at both low and high (10× low) concentrations, as listed in Supporting Table S1. Analytes were extracted from fortified whole blood, and the peak area detector counts were recorded and compared to the peak area detector counts of neat stocks (i.e., analyte in 1:1 (v/v) MeOH:H_2_O solution). Recovery was found by dividing the peak area detector counts of an extracted sample (“peak area extracted analyte”) by the neat stock (“peak area neat analyte”) at the same concentration using eq 2. This was repeated for each replicate and averaged, and the standard deviation was determined. The analyte recovery is reported as an average and a standard deviation.
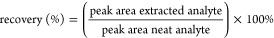
2

#### 2.6.6 Precision

Measurement precision described how close replicate measurements are and can be found from the average and standard deviation of measured peak area detector counts. Precision of each extracted analyte measurement, including both within run and between run data, was calculated at the low and high (10× low) concentrations using eq 3. While quantitative methods should be assessed for both within run and between run precision, this work considered an aggregate of the data and found one precision result for each analyte at low and high concentrations.
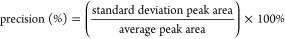
3

## 3 Results

A total of 35 analytes (all of the NSC’s Tier I drugs of abuse list^6^) were analyzed and validated using the method outlined above. Limits of detection were administratively set to align with the low concentrations considered here. To prevent repetition, results are presented as analytes with LODs greater than expected where the expected LOD is displayed in parentheses. These analytes are amphetamine (10 ng/mL), lorazepam (10 ng/mL), oxymorphone (10 ng/mL), Δ^9^-tetrahydrocannabinol (Δ^9^-THC, 5 ng/mL), and 11-hydroxy-Δ^9^-tetrahydrocannabinol (OH-Δ^9^-THC, 5 ng/mL). Amphetamine and lorazepam were not reliably detected at 10 ng/mL but were detected at 100 ng/mL. Oxymorphone, Δ^9^-THC, and OH-Δ^9^-THC were not reliably detected at the low and high concentrations considered here. Carryover was never detected for any analyte. Interference was not detected for endogenous or exogenous substances alongside any analyte. A list of exogenous substances considered as potential interferents for each analyte is detailed in Supporting Table S3.

Method performance results for analyte recovery, ion suppression or enhancement, and precision are summarized in Table 1. Ion suppression or enhancement for both low and high concentrations varied depending on compound, as displayed graphically in Supporting Figure S1. At low concentrations, ionization ranged from 30.1% (3,4-methylenedioxymethamphetamine, MDMA) to 267.6% (meprobamate) and averaged 110.5%. At high concentrations, ionization ranged from 8.6% (morphine) to 383.5% (6-acetylmorphine) and averaged 93.6%. Some analytes also displayed significant within-analyte ionization variability, represented by the standard deviation (and error bars in Supporting Figure S1). Analyte recovery also varied, as displayed graphically in Supporting Figure S2. At low concentrations, the recovery ranged from 8.5% (6-acetylmorphine, 6-AM) to 330.5% (oxycodone), averaging 105.3%. At high concentrations, recovery ranged from 9.4% (morphine) to 127.5% (alprazolam), averaging 82.7%. Some analytes also displayed significant within-analyte recovery variability (i.e., standard deviation in Table 1 and error bars in Supporting Figure S2). As another measure of measurement variability within analytes, precision is also listed in Table 1. At low concentrations, the precision ranged from 4.7% (7-aminoclonazepam) to 135.2% (clonazepam) and averaged 36.5%. At high concentrations, the precision ranged from 0.9% (tramadol) to 59.0% (meprobamate), averaging 21.7%.

Authentic suspected impaired driver and postmortem specimens (*N* = 166) were considered here. Spectral library matching was limited to the analytes considered in this work (NSC’s Tier I drugs and metabolites). Results are reported as the number of times each analyte was presumptively identified and a prevalence rate within the cohort of specimens, see Table 2. The most commonly identified analyte was COOH-Δ^9^-THC, occurring in 52 (31.3%) of specimens considered here. Despite lower prevalence rates, Δ^9^-THC (*N* = 3, 1.8%) and OH-Δ^9^-THC (*N* = 19, 11.4%) were also identified. This is despite the limitations described above in reliably detecting these cannabinoids at lower concentrations. Presumably, the cannabinoid concentrations observed in suspected impaired drivers and postmortem specimens are greater than the high concentration (50 ng/mL) considered in this work. The next most common analyte was fentanyl, occurring in 40 (24.1%) of the included specimens. Beyond that were methamphetamine (*N* = 36, 21.7%) and amphetamine (*N* = 32, 19.3%) followed by benzoylecgonine (*N* = 22, 13.3%) and cocaine (*N* = 20, 12.0%).

**Table 2:** Analysis of Authentic Suspected Impaired Driver and Postmortem Specimens (*N* = 166) for the 35 NSC Tier I Drugs and Metabolites Considered in This Work^a^

analyte	number of positive specimens	prevalence in specimens, %
stimulants		
methamphetamine	36	21.7
amphetamine	32	19.3
cocaine	20	12.0
cocaethylene	7	4.2
benzoylecgonine	22	13.3
MDMA	1	0.6
MDA	0	0.0
depressants		
carisoprodol	0	0.0
meprobamate	0	0.0
zolpidem	1	0.6
benzodiazepines		
7-aminoclonazepam	3	1.8
α-hydroxyalprazolam	0	0.0
alprazolam	5	3.0
clonazepam	4	2.4
lorazepam	1	0.6
diazepam	4	2.4
nordiazepam	5	3.0
oxazepam	2	1.2
temazepam	3	1.8
narcotic analgesics		
codeine	1	0.6
6-AM	0	0.0
hydrocodone	3	1.8
hydromorphone	1	0.6
morphine	5	3.0
oxycodone	3	1.8
oxymorphone	0	0.0
fentanyl	40	24.1
buprenorphine	1	0.6
norbuprenorphine	4	2.4
methadone	8	4.8
tramadol	6	3.6
*O*-desmethyltramadol	2	1.2
*cannabinoids*		
Δ^9^-THC	3	1.8
OH-Δ^9^-THC	19	11.4
COOH-Δ^9^-THC	52	31.3

a Results include the number of times each analyte was presumptively identified and the calculated prevalence rate within the cohort of authentic specimens.

A limitation of this work is that we still rely on in-house spectral library matching for identification. Relying on an in-house spectral library is sufficient for common compounds or those that have reference standards available but insufficient for novel or emerging compounds. This method also does not address structural analogues or isomers (such as (+)- versus (−)-methamphetamine) that cannot be distinguished via retention times or fragmentation patterns in the MS/MS data. Additionally, the processed sample stability and carryover from concentrations higher than what was included here are outside the scope of this work.

## 4 Discussion

The broad extraction technique was designed to isolate many different compounds regardless of their differences in acid–base characteristics, polarity, and lipophilicity at low concentrations. Notably, most drugs were detectable at or below the recommended low-concentration cutoffs displayed in Supporting Table S1. However, there were a few compounds for which this method did not perform well. Amphetamine and lorazepam were not reliably detected at 10 ng/mL but could be detected at higher concentrations. Oxymorphone, Δ^9^-THC, and OH-Δ^9^-THC were not detected at up to 10× their low-concentration cutoffs listed in Supporting Table S1. Arguably, LODs are the most important measure of a qualitative method, as they represent which analytes will be identified at low concentrations. As stated above, not all analytes included the recommended low-concentration cutoffs. Screening low-concentration cutoffs were given for 12 of the 35 analytes: methamphetamine, amphetamine, benzoylecgonine, carisoprodol, zolpidem, burprenorphine, fentanyl, methadone, morphine, oxycodone, tramadol, and COOH-Δ^9^-THC. Confirmation low-concentration cutoffs were given for all analytes except for α-hydroxyalprazolam. Given the intended purpose of this assay, the LODs for 30 of the 35 Tier I analytes were acceptable. It is critical that those seeking to utilize this or similar assays realize and account for the limitations for some analytes, as is described here.

Ion suppression or enhancement was evaluated by comparing the peak area detector counts of an extracted analyte to those of the neat analyte. When the data were broken down into drug classes (i.e., stimulants, depressants, benzodiazepines, and narcotic analgesics), some trends were observed. Narcotic analgesics had an average ionization of 100.7% at low concentrations and 90.1% at high concentrations. Stimulants had many compounds with ionizations above 100%, particularly at low concentrations. The average ionization at low concentrations was 130.6, and 94.8% at high concentrations. It was also observed that ionization was closer to 100% and less variable at the higher concentrations compared to the lower concentrations, as shown in Supporting Figure S1. An explanation for this could be that at lower concentrations, effects from ionization are more prominent. At higher concentrations, the instrument’s response from the analytes is greater, which could diminish matrix effects.

To assess analyte recovery, postextraction addition was the chosen method. Analyte recoveries hovered mostly around 100% (meaning most analyte was recovered during sample preparation). The large standard deviations highlight the variability of the results. Variable recoveries were expected when developing a method that could accommodate a wide range of analytes. In addition, working with a complex matrix such as whole blood with minimal cleanup before extraction leads to variable results as shown by the large standard deviation. Furthermore, the use of a single internal standard (here, (±)-warfarin-d5) minimized the costs and complexity of the sample preparation procedure but does not adequately correct for variations in the sample preparation and data acquisition steps. It was also observed that recoveries of over 100% tended to occur alongside ionizations greater than 100%. As this is a qualitative method, low recovery percentages were tolerated if the target analytes were reproducibly detected at low concentrations, which was true for most compounds.

Cannabinoids were particularly challenging to extract and analyze, as expected from their lipophilic nature.^35^ Their chemical properties are quite different from the majority of other, more polar drugs of abuse, and as such, extraction and data acquisition are more challenging to tailor.^22^ The interaction between the cannabinoid compounds and the lipid cleanup plate may have played a role in their results. Because cannabinoids are quite lipophilic, they likely behave more like lipids and thus are not eluted through the plate as efficiently as the other more polar compounds. As a result, neither Δ^9^-THC nor OH-Δ^9^-THC was extracted or ionized efficiently for detection at the recommended low concentrations nor at 10× that. However, COOH-Δ^9^-THC was successfully extracted and analyzed at both low and high concentrations. It is worth noting that in this work, negative-ion mode only included three analytes, two of which were not reliably detected (Δ^9^-THC and OH-Δ^9^-THC) at either 5 or 50 ng/mL. Despite this well-documented limitation of DIA approaches,^21^ the ability to detect COOH-Δ^9^-THC at low concentrations (5 ng/mL) is adequate to flag specimens for confirmatory cannabinoid quantification. This is exemplified by the prevalence of COOH-Δ^9^-THC (*N* = 52, 31.3%) in authentic suspected impaired driver and postmortem specimens. Furthermore, Δ^9^-THC (*N* = 3, 1.8%) and OH-Δ^9^-THC (*N* = 19, 11.4%) were detected in authentic suspected impaired driver and postmortem specimens. Presumably, this is due to a much higher concentration within the biological specimens than what was considered here.

Three other compounds were found to be difficult to reliably detect at low concentrations: amphetamine, lorazepam, and oxymorphone. Initially, it was suspected that interference from similar compounds, such as methamphetamine, other benzodiazepines, and/or other opiates, could be the source. This possibility was explored by analyzing each compound individually and in combination with potential interferents. It was found that the detection of these analytes was not improved even when evaluated individually; thus, the conclusion is that these compounds are not detected reliably at the low concentrations considered here. However, amphetamine was presumptively identified in 32 (19.3%) and lorazepam in 1 (0.6%) authentic suspected impaired driver and postmortem specimens. As with the cannabinoids, we assume these biological specimens contained much higher concentrations of analytes than our low- and high-concentration fortifications. Oxymorphone was not detected in any of the 166 authentic specimens considered here.

## 5 Conclusions

Our goal of assessing the performance of an untargeted sample preparation and data acquisition method for a wide range of forensically relevant analytes simultaneously was achieved by using SLE and HRMS. The method outlined was fast and involved few steps while requiring minimal sample/solvent volume. The preparation with the lipid cleanup was quick and utilized only 100 μL of blood which is ideal for routine toxicological screens but was not suitable for low concentrations of cannabinoids beyond COOH- Δ^9^-THC. By using HRMS, most Tier I analytes were identified at or below the recommended low concentration cutoffs including within a cohort of 166 authentic suspected impaired driver and postmortem specimens.

## Supporting Information Available

Summary of recommended screening low concentration cutoffs for blood specimens and associated concentrations considered in this work; relevant liquid chromatography (LC) and high-resolution mass spectrometry (HRMS) details (i.e., expected retention time (RT), neutral mass, exact mass, and fragments) used for identification of analytes; exogenous interferents considered for each analyte; and ion suppression or enhancement and analyte recovery data at low and high concentrations (PDF)
