# Supplementary material for: Screening for Forensically Relevant Drugs Using Data-Independent High-Resolution Mass Spectrometry
Source: Chem Res Toxicol. 2024 Apr 4;37(4):571–9. doi: 10.1021/acs.chemrestox.3c00379 (PMC11022238; doi:10.1021/acs.chemrestox.3c00379)
Supplement: Supplementary file 1 — tx3c00379_si_001.pdf [file tx3c00379_si_001.pdf]

Supplementary Information

Screening for forensically relevant drugs using data-independent high-resolution mass spectrometry

Maia N. Bates[†] [‡], Abby E. Helm[§], and Heather M. Barkholtz[‡] [§] *

[†] Department of Chemistry, College of Letters and Science, University of Wisconsin-Madison, 1101 University Avenue, Madison, WI 53706, USA

[‡] Forensic Toxicology Section, Environmental Health Division, Wisconsin State Laboratory of Hygiene, 2601 Agriculture Drive, Madison, WI 53718, USA

[§] Pharmaceutical Sciences Division, School of Pharmacy, University of Wisconsin-Madison, 777 Highland Avenue, Madison, WI 53705, USA

* hbarkholtz@wisc.edu, 608-890-1967

Table of Contents

Table S1. Summary of recommended screening low concentration cutoffs for blood specimens and associated concentrations considered in this work………………………………………………………………………. **S3**

Table S2. Relevant liquid chromatography (LC) and high-resolution mass spectrometry (HRMS) details (i.e., expected retention time (RT), neutral mass, exact mass, and fragments) used for identification of analytes…….. **S4**

Table S3. Exogenous interferents considered for each analyte……………………………………………………….. **S6**

Figure S1. Ion suppression or enhancement data for each analyte at low and high concentrations….. **S9**

Figure S2. Analyte recovery data for each analyte at low and high concentrations…………………………… **S9**

Table S1. The National Safety Council's (NSC's) Tier I drugs of abuse and metabolites list of analytes and recommended screening and confirmation assay low concentration cutoff for blood samples. Also included are the low and high (10x low) concentrations of each analyte considered in this work.

Analyte	Screen (ng/mL)	Confirm (ng/mL)	This work (ng/mL)
Methamphetamine	20	20	10 & 100
Amphetamine	20	20	10 & 100
Cocaine	-	10	20 & 200
Benzoylecgonine	50	50	50 & 500
Cocaethylene	-	10	10 & 100
3,4-Methylenedioxy methamphetamine (MDMA)	-	20	10 & 100
3,4-Methylenedioxyamphetamine (MDA)	-	20	10 & 100
Carisoprodol	1,000	1,000	1,000 & 10,000
Meprobamate	-	500	2,000 & 20,000
Zolpidem	10	10	10 & 100
7-Aminoclonazepam	-	10	10 & 100
α-Hydroxyalprazolam	-	-	10 & 100
Alprazolam	-	10	10 & 100
Clonazepam	-	10	10 & 100
Lorazepam	-	10	10 & 100
Diazepam	-	20	10 & 100
Nordiazepam	-	20	10 & 100
Oxazepam	-	20	10 & 100
Temazepam	-	20	10 & 100
Codeine	-	10	10 & 100
6-Acetylmorphine (6-AM)	-	5	4 & 40
Buprenorphine	1	0.5	1 & 10
Norbuprenorphine	-	1	1 & 10
Fentanyl	1	0.5	1 & 10
Hydrocodone	-	10	10 & 100
Hydromorphone	-	5	10 & 100
Methadone	50	20	20 & 200
Morphine	10	10	10 & 100
Oxycodone	10	10	10 & 100
Oxymorphone	-	5	10 & 100
Tramadol	100	50	50 & 500
O-desmethyltramadol	-	50	50 & 500
Δ^9-tetrahydrocannabinol (Δ^9-THC)	-	1	10 & 100
11-hydroxy-Δ^9-tetrahydrocannabinol (OH-Δ^9-THC)	-	1	10 & 100
11-nor-9-carboxy-Δ^9-tetrahydrocannabinol (COOH-Δ^9-THC)	10	5	5 & 50

Table S2. Relevant liquid chromatography (LC) and high-resolution mass spectrometry (HRMS) details used for identification of analytes. Details include expected retention time (RT), neutral mass, exact mass, and fragments.

Analyte	ESI	RT (min)	Neutral mass (Da)	Exact mass (m/z)	Fragment 1 (m/z)	Fragment 2 (m/z)	Fragment 3 (m/z)	Fragment 4 (m/z)
Methamphetamine	+	2.47	149.12045	150.1269	91.0533	119.0847		
Amphetamine	+	2.18	135.10480	136.1118	91.0534	119.0850		
Cocaine	+	4.50	303.14706	304.1539	182.1169	82.0643	105.0327	150.0907
Cocaethylene	+	5.50	317.06271	318.1696	196.1328	82.0644	150.0908	168.1014
Benzoylecgonine	+	2.95	289.13141	290.1384	168.1014	105.0327		
3,4-Methylenedioxy methamphetamine (MDMA)	+	1.46	193.11028	194.1170	105.0692	163.0748	135.0435	
3.4-Methylenedioxy amphetamine (MDA)	+	2.22	179.09463	180.1013	135.0434	133.0643	163.0749	
Carisoprodol	+	8.32	260.17361	261.1806	97.1005	176.1278	158.1171	
Meprobamate	+	4.89	218.12666	219.1339	158.1170	97.1005		
Zolpidem	+	4.78	307.16846	308.1752	235.1225	236.1287	263.1174	
7-Aminoclonazepam	+	3.78	285.06680	286.0742	250.0975	222.1026	121.0761	
α-Hydroxyalprazolam	+	7.70	324.07779	325.0851	307.0732	297.0660	295.0742	204.0314
Alprazolam	+	8.51	308.08287	309.0896	281.0711	274.1208		
Clonazepam	+	8.23	315.04107	316.0477	270.0549	241.0519	214.0410	
Lorazepam	+	8.26	320.01193	321.0189	275.0135	229.0525	138.0098	
Diazepam	+	10.5	284.07164	285.0783	193.0878	154.0410	222.1144	
Nordiazepam	+	8.97	270.05599	271.0627	140.0253	208.0986	165.0206	243.0674
Oxazepam	+	7.92	286.05091	287.0579	241.0524	269.0524	231.0682	
Temazepam	+	9.12	300.06656	301.0732	255.0678	193.0880	228.0570	
Codeine	+	1.70	299.15214	300.1587	215.1057	225.0903	282.1483	
6-Acetylmorphine (6-AM)	+	2.10	327.14706	328.1544	165.0696	211.0755	193.0648	268.1324
Hydrocodone	+	2.32	299.15214	300.1591	199.0748	171.0798	241.0859	
Hydromorphone	+	1.19	285.13649	286.1432	185.0591	227.0704	199.0748	
Morphine	+	1.10	285.13649	286.1432	165.0683	201.0902		
Oxycodone	+	2.09	315.14706	316.1536	298.1430	241.1075	256.1324	
Oxymorphone	+	1.12	301.13141	302.1382	284.1277	227.0921	242.1167	
Fentanyl	+	6.18	336.22016	337.2270	188.1427	105.0691	216.1379	
Buprenorphine	+	6.97	467.30356	468.3107	414.2642	396.2176		
Norbuprenorphine	+	5.08	413.25661	414.2637	396.2510			
Methadone	+	8.44	309.20926	310.2165	265.1587	105.0327	219.1163	
Tramadol	+	3.87	263.18853	264.1949	58.0641	246.1847		
O-desmethyltramadol	+	2.30	249.17288	250.1795	58.0642	232.1691		
Δ^9-tetrahydrocannabinol (Δ^9-THC)	-	5.58	314.22458	313.2228	245.1547	229.1234	191.1078	179.1078

11-hydroxy-Δ^9-tetrahydrocannabinol (OH-Δ^9-THC)	-	4.92	330.21949	329.2119	311.2017	281.1547	268.1469	267.1391
11-nor-9-carboxy-Δ^9-tetrahydrocannabinol (COOH-Δ^9-THC)	-	4.91	344.19876	343.1855	299.2024	245.1548	191.1079	179.1080
Warfarin-D5	±	10.28	313.13624	314.1437	256.1017			

Table S3. Exogenous interferents considered for each analyte.

Analyte	Interferents considered
Methamphetamine	Amphetamine, benzoylecgonine, clonazepam, cocaine, cocaethylene, codeine, doxylamine, hydromorphone, MDA, MDMA, midazolam, norbuprenorphine, olanzapine, primidone, phentermine, sertraline, zolpidem
Amphetamine	Acetylfentanyl, alprazolam, benzoylecgonine, buprenorphine, chlorpheniramine, cocaine, cocaethylene, cyclobenzaprine, fluoxetine, hydrocodone, lorazepam, MDA, MDMA, methamphetamine, phencyclidine (PCP), phentermine, promethazine, triazolam
Cocaine	Amphetamine, benzoylecgonine, clonazepam, cocaethylene, codeine, doxylamine, hydromorphone, MDA, MDMA, methamphetamine, midazolam, norbuprenorphine, olanzapine, phentermine, primidone, sertraline, zolpidem
Benzoylecgonine	Amphetamine, benzoylecgonine, carisoprodol, citalopram, cocaine, cocaethylene, ephedrine, estazolam, MDA, MDMA, methamphetamine, oxazepam, oxymorphone, paroxetine, phentermine, tapentadol
Cocaethylene	Amphetamine, benzoylecgonine, chlordiazepoxide, cocaine, cocaethylene, dextromethorphan, diazepam, fentanyl, MDA, MDMA, methadone, methamphetamine, morphine, nordiazepam, oxycodone, phentermine, topiramate, venlafaxine
3,4-Methylenedioxy methamphetamine (MDMA)	Acetylfentanyl, alprazolam, amphetamine, benzoylecgonine, buprenorphine, chlorpheniramine, cyclobenzaprine, cocaine, cocaethylene, fluoxetine, hydrocodone, lorazepam, MDA, methamphetamine, phencyclidine (PCP), phentermine, promethazine, triazolam
3,4-Methylenedioxyamphetamine (MDA)	Amphetamine, benzoylecgonine, clonazepam, cocaine, cocaethylene, codeine, doxylamine, hydromorphone, MDMA, methamphetamine, midazolam, norbuprenorphine, olanzapine, phentermine, primidone, sertraline, zolpidem
Carisoprodol	Benzoylecgonine, citalopram, ephedrine, estazolam, meprobamate, oxazepam, oxymorphone, paroxetine, tapentadol, zolpidem
Meprobamate	Carisoprodol, diphenhydramine, duloxetine, flualprazolam, ketamine, levetiracetam, mitragynine, morphine, phentermine, temazepam, trazadone, zolpidem
Zolpidem	Carisoprodol, clonazepam, cocaine, codeine, doxylamine, hydromorphone, MDA, meprobamate, methamphetamine, midazolam, norbuprenorphine, olanzapine, primidone, sertraline
7-Aminoclonazepam	Alprazolam, clonazepam, diazepam, α-hydroxyalprazolam, lorazepam, nordiazepam, oxazepam, temazepam
α-Hydroxyalprazolam	7-aminoclonazepam, alprazolam, clonazepam, diazepam, lorazepam, nordiazepam, oxazepam, temazepam
Alprazolam	7-aminoclonazepam, acetylfentanyl, amphetamine, buprenorphine, chlorpheniramine, clonazepam, cyclobenzaprine, diazepam, fluoxetine, hydrocodone, α-hydroxyalprazolam, lorazepam, MDA, MDMA, nordiazepam, oxazepam, phencyclidine (PCP), promethazine, temazepam, triazolam
Clonazepam	7-aminoclonazepam, alprazolam, cocaine, codeine, diazepam, doxylamine, α-hydroxyalprazolam, hydromorphone, lorazepam, MDA, methamphetamine, midazolam, norbuprenorphine, nordiazepam, olanzapine, oxazepam, primidone, sertraline, temazepam, zolpidem

Lorazepam	7-aminoclonazepam, acetylfentanyl, alprazolam, amphetamine, buprenorphine, chlorpheniramine, clonazepam, cyclobenzaprine, diazepam, fluoxetine, hydrocodone, α-hydroxyalprazolam, MDMA, nordiazepam, oxazepam, phencyclidine (PCP), promethazine, temazepam, triazolam
Diazepam	7-aminoclonazepam, alprazolam, chlordiazepoxide, clonazepam, cocaethylene, dextromethorphan, fentanyl, α-hydroxyalprazolam, lorazepam, methadone, morphine, nordiazepam, oxazepam, oxycodone, temazepam, topiramate, venlafaxine
Nordiazepam	7-aminoclonazepam, alprazolam, chlordiazepoxide, clonazepam, cocaethylene, dextromethorphan, diazepam, fentanyl, α-hydroxyalprazolam, lorazepam, methadone, morphine, oxazepam, oxycodone, topiramate, venlafaxine
Oxazepam	7-aminoclonazepam, alprazolam, benzoylecgonine, carisoprodol, citalopram, clonazepam, diazepam, ephedrine, estazolam, α-hydroxyalprazolam, lorazepam, nordiazepam, oxymorphone, paroxetine, tapentadol, temazepam
Temazepam	7-aminoclonazepam, alprazolam, clonazepam, diazepam, diphenhydramine, duloxetine, flualprazolam, α-hydroxyalprazolam, ketamine, levetiracetam, lorazepam, meprobamate, mitragynine, morphine, nordiazepam, oxazepam, phentermine, temazepam, trazadone
Codeine	6-acetylmorphine, buprenorphine, clonazepam, cocaine, doxylamine, fentanyl, hydrocodone, hydromorphone, MDA, methadone, methamphetamine, midazolam, morphine, norbuprenorphine, O-desmethyltramadol, olanzapine, oxycodone, oxymorphone, primidone, sertraline, tramadol, zolpidem
6-Acetylmorphine (6-AM)	6-acetylmorphine, Buprenorphine, fentanyl, hydrocodone, hydromorphone, methadone, morphine, norbuprenorphine, O-desmethyltramadol, oxycodone, oxymorphone, tramadol
Buprenorphine	6-acetylmorphine, acetylfentanyl, alprazolam, amphetamine, chlorpheniramine, cyclobenzaprine, fentanyl, fluoxetine, hydrocodone, hydromorphone, lorazepam, MDMA, methadone, morphine, norbuprenorphine, O-desmethyltramadol, oxycodone, oxymorphone, phencyclidine (PCP), promethazine, triazolam, tramadol
Norbuprenorphine	6-acetylmorphine, buprenorphine, clonazepam, cocaine, codeine, doxylamine, fentanyl, hydrocodone, hydromorphone, MDA, methadone, methamphetamine, midazolam, morphine, O-desmethyltramadol, olanzapine, oxycodone, oxymorphone, primidone, sertraline, tramadol, zolpidem
Fentanyl	6-acetylmorphine, buprenorphine, chlordiazepoxide, cocaethylene, dextromethorphan, diazepam, hydrocodone, hydromorphone, methadone, morphine, norbuprenorphine, nordiazepam, O-desmethyltramadol, oxycodone, oxymorphone, topiramate, tramadol, venlafaxine
Hydrocodone	6-acetylmorphine, acetylfentanyl, alprazolam, amphetamine, buprenorphine, chlorpheniramine, cyclobenzaprine, fentanyl, fluoxetine, hydromorphone, lorazepam, MDMA, methadone, morphine, norbuprenorphine, O-desmethyltramadol, oxycodone, oxymorphone, phencyclidine (PCP), promethazine, triazolam, tramadol
Hydromorphone	6-acetylmorphine, buprenorphine, clonazepam, cocaine, codeine, doxylamine, fentanyl, hydrocodone, MDA, methamphetamine,

	methadone, midazolam, morphine, norbuprenorphine, O-desmethyltramadol, olanzapine, oxycodone, oxymorphone, primidone, sertraline, tramadol, zolpidem
Methadone	6-acetylmorphine, buprenorphine, chlordiazepoxide, cocaethylene, dextromethorphan, diazepam, fentanyl, hydrocodone, hydromorphone, morphine, norbuprenorphine, nordiazepam, O-desmethyltramadol, oxycodone, oxymorphone, topiramate, tramadol, venlafaxine
Morphine	6-acetylmorphine, buprenorphine, diphenhydramine, duloxetine, fentanyl, flualprazolam, hydrocodone, hydromorphone, ketamine, levetiracetam, meprobamate, methadone, mitragynine, norbuprenorphine, O-desmethyltramadol, oxycodone, oxymorphone, phentermine, temazepam, tramadol, trazadone
Oxycodone	6-acetylmorphine, buprenorphine, chlordiazepoxide, cocaethylene, dextromethorphan, diazepam, fentanyl, hydrocodone, hydromorphone, methadone, morphine, norbuprenorphine, nordiazepam, O-desmethyltramadol, oxymorphone, topiramate, tramadol, venlafaxine
Tramadol	6-acetylmorphine, atropine, benztropine, brorphine, Buprenorphine, etizolam, fentanyl, gabapentin, hydrocodone, hydromorphone, isotonitazene, lidocaine, methadone, mirtazapine, morphine, norbuprenorphine, O-desmethyltramadol, oxycodone, oxymorphone
O-desmethyltramadol	6-acetylmorphine, buprenorphine, fentanyl, hydrocodone, hydromorphone, methadone, morphine, norbuprenorphine, oxycodone, oxymorphone, tramadol
11-nor-9-carboxy-Δ^9-tetrahydrocannabinol (COOH-Δ^9-THC)	Δ^9-tetrahydrocannabinol, 11-hydroxy-Δ^9-tetrahydrocannabinol

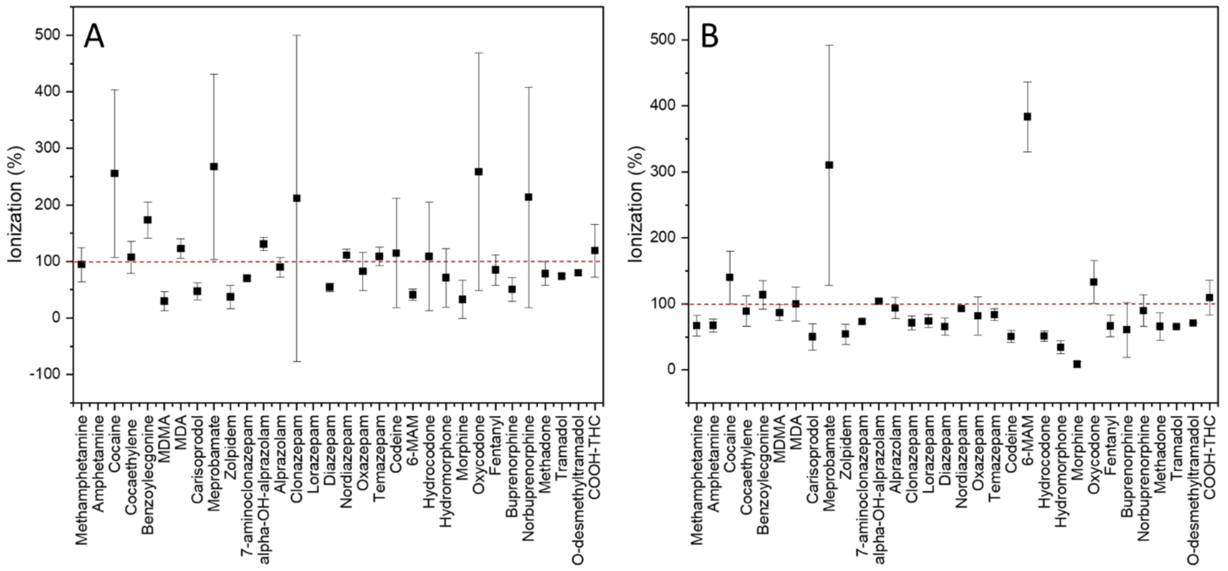

Figure S1. Ion suppression or enhancement data (solid squares) for all analytes at low concentration (**A**) and high concentration (**B**). A dotted red line is included at 100% ionization to aid the reader in identifying where optimal (i.e., minimal ion suppression (< 100%) or enhancement (> 100%) results appear. Standard deviation is displayed as error bars above and below the average ionization result for each analyte. Results for oxymorphone, Δ^9-THC, and OH-Δ^9-THC were omitted from this figure as they were not reliably detected at the concentrations considered in this work.

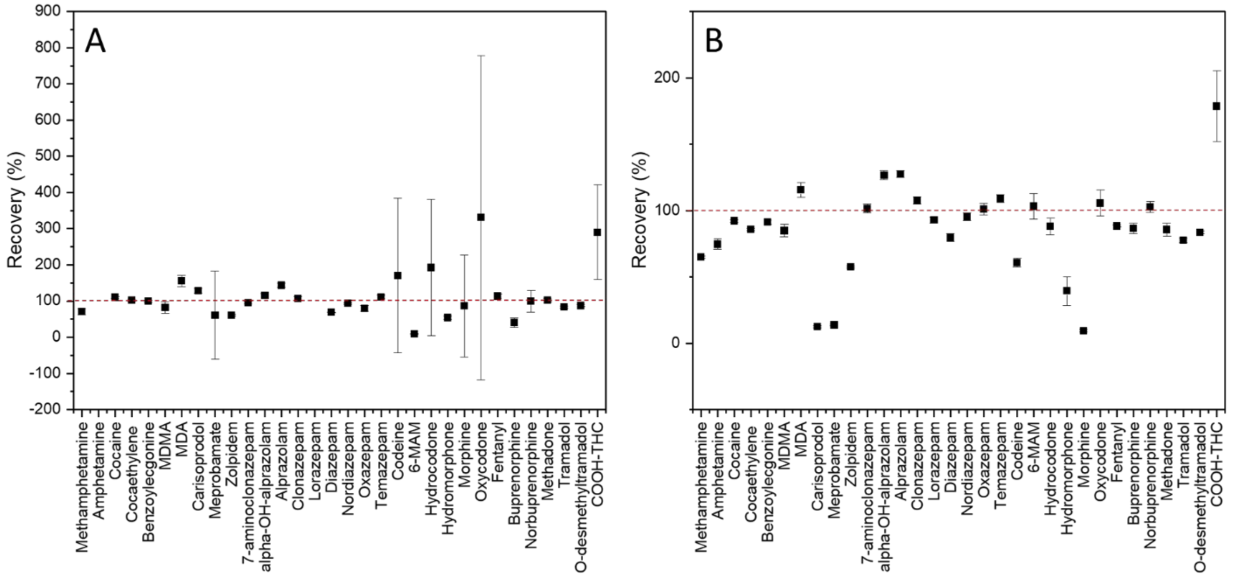

Figure S2. Analyte recovery data (solid squares) for all analytes at low concentration (**A**) and high concentration (**B**). A dotted red line is included at 100% recovery to aid the reader in identifying where optimal results will appear. Standard deviation is displayed as error bars above and below the average recovery result for each analyte. Results for oxymorphone, Δ^9-THC, and OH-Δ^9-THC were omitted from this figure as they were not reliably detected at the concentrations considered in this work. Please note that the y-axis scale differs in panels A and B.
